# Supplementary material for: COZOID: contact zone identifier for visual analysis of protein-protein interactions
Source: BMC Bioinformatics. 2018 Apr 6;19:125. doi: 10.1186/s12859-018-2113-6 (PMC5889581; doi:10.1186/s12859-018-2113-6)
Supplement: Supplementary file 2 — Software build. Executable binary file of the software tool. (ZIP 118,784 kb) [file 12859_2018_2113_MOESM2_ESM.zip › cozoid/java/docs/beansbinding-1.2.1-doc.zip!/org/jdesktop/el/MapELResolver.html]

MapELResolver (Beans Binding)


---


|  |  |  |
| --- | --- | --- |
| **Overview** \| **Package** \| **Class** \| **Use** \| **Tree** \| **Deprecated** \| **Index** \| **Help** | |  |
| **PREV CLASS**   **NEXT CLASS** | **FRAMES**    **NO FRAMES**     **All Classes** |  |
| SUMMARY: NESTED \| FIELD \| CONSTR \| METHOD | DETAIL: FIELD \| CONSTR \| METHOD |  |


---


## org.jdesktop.el Class MapELResolver

```
java.lang.Object
  org.jdesktop.el.ELResolver
      org.jdesktop.el.MapELResolver
```

---

``` public class MapELResolver extends ELResolver ```

Defines property resolution behavior on instances of `Map`.

This resolver handles base objects of type `java.util.Map`.
It accepts any object as a property and uses that object as a key in
the map. The resulting value is the value in the map that is associated with
that key.

This resolver can be constructed in read-only mode, which means that
`isReadOnly` will always return `true` and
`setValue(org.jdesktop.el.ELContext, java.lang.Object, java.lang.Object, java.lang.Object)` will always throw
`PropertyNotWritableException`.

`ELResolver`s are combined together using
`CompositeELResolver`s, to define rich semantics for evaluating
an expression. See the javadocs for `ELResolver` for details.

**Since:**
:   JSP 2.1

**See Also:**: `CompositeELResolver`, `ELResolver`, `Map`

---

**Field Summary**

**Fields inherited from class org.jdesktop.el.ELResolver**

`RESOLVABLE_AT_DESIGN_TIME, TYPE`


**Constructor Summary**

`MapELResolver()`
  
          Creates a new read/write `MapELResolver`.

`MapELResolver(boolean isReadOnly)`
  
          Creates a new `MapELResolver` whose read-only status is
determined by the given parameter.


| **Method Summary** | |
| --- | --- |
| `java.lang.Class<?>` | `getCommonPropertyType(ELContext context, java.lang.Object base)`             If the base object is a map, returns the most general type that this resolver accepts for the `property` argument. |
| `java.util.Iterator<java.beans.FeatureDescriptor>` | `getFeatureDescriptors(ELContext context, java.lang.Object base)`             If the base object is a map, returns an `Iterator` containing the set of keys available in the `Map`. |
| `java.lang.Class<?>` | `getType(ELContext context, java.lang.Object base, java.lang.Object property)`             If the base object is a map, returns the most general acceptable type for a value in this map. |
| `java.lang.Object` | `getValue(ELContext context, java.lang.Object base, java.lang.Object property)`             If the base object is a map, returns the value associated with the given key, as specified by the `property` argument. |
| `boolean` | `isReadOnly(ELContext context, java.lang.Object base, java.lang.Object property)`             If the base object is a map, returns whether a call to `setValue(org.jdesktop.el.ELContext, java.lang.Object, java.lang.Object, java.lang.Object)` will always fail. |
| `void` | `setValue(ELContext context, java.lang.Object base, java.lang.Object property, java.lang.Object val)`             If the base object is a map, attempts to set the value associated with the given key, as specified by the `property` argument. |

**Methods inherited from class java.lang.Object**

`clone, equals, finalize, getClass, hashCode, notify, notifyAll, toString, wait, wait, wait`

**Constructor Detail**

### MapELResolver

```
public MapELResolver()
```

:   Creates a new read/write `MapELResolver`.

---


### MapELResolver

```
public MapELResolver(boolean isReadOnly)
```

:   Creates a new `MapELResolver` whose read-only status is
    determined by the given parameter.

    **Parameters:**: `isReadOnly` - `true` if this resolver cannot modify maps; `false` otherwise.


**Method Detail**

### getType

```
public java.lang.Class<?> getType(ELContext context,
                                  java.lang.Object base,
                                  java.lang.Object property)
```

:   If the base object is a map, returns the most general acceptable type
    for a value in this map.

    If the base is a `Map`, the `propertyResolved`
    property of the `ELContext` object must be set to
    `true` by this resolver, before returning. If this property
    is not `true` after this method is called, the caller
    should ignore the return value.

    Assuming the base is a `Map`, this method will always
    return `Object.class`. This is because `Map`s
    accept any object as the value for a given key.

    :   **Specified by:**: `getType` in class `ELResolver`
    :   **Parameters:**: `context` - The context of this evaluation.: `base` - The map to analyze. Only bases of type `Map` are handled by this resolver.: `property` - The key to return the acceptable type for. Ignored by this resolver. **Returns:**: If the `propertyResolved` property of `ELContext` was set to `true`, then the most general acceptable type; otherwise undefined. **Throws:**: `java.lang.NullPointerException` - if context is `null`: `ELException` - if an exception was thrown while performing the property or variable resolution. The thrown exception must be included as the cause property of this exception, if available.

---


### getValue

```
public java.lang.Object getValue(ELContext context,
                                 java.lang.Object base,
                                 java.lang.Object property)
```

:   If the base object is a map, returns the value associated with the
    given key, as specified by the `property` argument. If the
    key was not found, `null` is returned.

    If the base is a `Map`, the `propertyResolved`
    property of the `ELContext` object must be set to
    `true` by this resolver, before returning. If this property
    is not `true` after this method is called, the caller
    should ignore the return value.

    Just as in `Map.get(java.lang.Object)`, just because `null`
    is returned doesn't mean there is no mapping for the key; it's also
    possible that the `Map` explicitly maps the key to
    `null`.

    :   **Specified by:**: `getValue` in class `ELResolver`
    :   **Parameters:**: `context` - The context of this evaluation.: `base` - The map to be analyzed. Only bases of type `Map` are handled by this resolver.: `property` - The key whose associated value is to be returned. **Returns:**: If the `propertyResolved` property of `ELContext` was set to `true`, then the value associated with the given key or `null` if the key was not found. Otherwise, undefined. **Throws:**: `java.lang.ClassCastException` - if the key is of an inappropriate type for this map (optionally thrown by the underlying `Map`).: `java.lang.NullPointerException` - if context is `null`, or if the key is null and this map does not permit null keys (the latter is optionally thrown by the underlying `Map`).: `ELException` - if an exception was thrown while performing the property or variable resolution. The thrown exception must be included as the cause property of this exception, if available.

---


### setValue

```
public void setValue(ELContext context,
                     java.lang.Object base,
                     java.lang.Object property,
                     java.lang.Object val)
```

:   If the base object is a map, attempts to set the value associated with
    the given key, as specified by the `property` argument.

    If the base is a `Map`, the `propertyResolved`
    property of the `ELContext` object must be set to
    `true` by this resolver, before returning. If this property
    is not `true` after this method is called, the caller
    can safely assume no value was set.

    If this resolver was constructed in read-only mode, this method will
    always throw `PropertyNotWritableException`.

    If a `Map` was created using
    `Collections.unmodifiableMap(java.util.Map extends K, ? extends V)`, this method must
    throw `PropertyNotWritableException`. Unfortunately,
    there is no Collections API method to detect this. However, an
    implementation can create a prototype unmodifiable `Map`
    and query its runtime type to see if it matches the runtime type of
    the base object as a workaround.

    :   **Specified by:**: `setValue` in class `ELResolver`
    :   **Parameters:**: `context` - The context of this evaluation.: `base` - The map to be modified. Only bases of type `Map` are handled by this resolver.: `property` - The key with which the specified value is to be associated.: `val` - The value to be associated with the specified key. **Throws:**: `java.lang.ClassCastException` - if the class of the specified key or value prevents it from being stored in this map.: `java.lang.NullPointerException` - if context is `null`, or if this map does not permit `null` keys or values, and the specified key or value is `null`.: `java.lang.IllegalArgumentException` - if some aspect of this key or value prevents it from being stored in this map.: `ELException` - if an exception was thrown while performing the property or variable resolution. The thrown exception must be included as the cause property of this exception, if available.: `PropertyNotWritableException` - if this resolver was constructed in read-only mode, or if the put operation is not supported by the underlying map.

---


### isReadOnly

```
public boolean isReadOnly(ELContext context,
                          java.lang.Object base,
                          java.lang.Object property)
```

:   If the base object is a map, returns whether a call to
    `setValue(org.jdesktop.el.ELContext, java.lang.Object, java.lang.Object, java.lang.Object)` will always fail.

    If the base is a `Map`, the `propertyResolved`
    property of the `ELContext` object must be set to
    `true` by this resolver, before returning. If this property
    is not `true` after this method is called, the caller
    should ignore the return value.

    If this resolver was constructed in read-only mode, this method will
    always return `true`.

    If a `Map` was created using
    `Collections.unmodifiableMap(java.util.Map extends K, ? extends V)`, this method must
    return `true`. Unfortunately, there is no Collections API
    method to detect this. However, an implementation can create a
    prototype unmodifiable `Map` and query its runtime type
    to see if it matches the runtime type of the base object as a
    workaround.

    :   **Specified by:**: `isReadOnly` in class `ELResolver`
    :   **Parameters:**: `context` - The context of this evaluation.: `base` - The map to analyze. Only bases of type `Map` are handled by this resolver.: `property` - The key to return the read-only status for. Ignored by this resolver. **Returns:**: If the `propertyResolved` property of `ELContext` was set to `true`, then `true` if calling the `setValue` method will always fail or `false` if it is possible that such a call may succeed; otherwise undefined. **Throws:**: `java.lang.NullPointerException` - if context is `null`: `ELException` - if an exception was thrown while performing the property or variable resolution. The thrown exception must be included as the cause property of this exception, if available.

---


### getFeatureDescriptors

```
public java.util.Iterator<java.beans.FeatureDescriptor> getFeatureDescriptors(ELContext context,
                                                                              java.lang.Object base)
```

:   If the base object is a map, returns an `Iterator`
    containing the set of keys available in the `Map`.
    Otherwise, returns `null`.

    The `Iterator` returned must contain zero or more
    instances of `FeatureDescriptor`. Each info object
    contains information about a key in the Map, and is initialized as
    follows:

    - displayName - The return value of calling the
      `toString` method on this key, or
      `"null"` if the key is `null`.
    - name - Same as displayName property.
    - shortDescription - Empty string
    - expert - `false`
    - hidden - `false`
    - preferred - `true`

    In addition, the following named attributes must be set in the
    returned `FeatureDescriptor`s:

    - `ELResolver.TYPE` - The return value of calling the `getClass()`
      method on this key, or `null` if the key is
      `null`.
    - `ELResolver.RESOLVABLE_AT_DESIGN_TIME` - `true`

    :   **Specified by:**: `getFeatureDescriptors` in class `ELResolver`
    :   **Parameters:**: `context` - The context of this evaluation.: `base` - The map whose keys are to be iterated over. Only bases of type `Map` are handled by this resolver. **Returns:**: An `Iterator` containing zero or more (possibly infinitely more) `FeatureDescriptor` objects, each representing a key in this map, or `null` if the base object is not a map. **See Also:**: `FeatureDescriptor`

---


### getCommonPropertyType

```
public java.lang.Class<?> getCommonPropertyType(ELContext context,
                                                java.lang.Object base)
```

:   If the base object is a map, returns the most general type that
    this resolver accepts for the `property` argument.
    Otherwise, returns `null`.

    Assuming the base is a `Map`, this method will always
    return `Object.class`. This is because `Map`s
    accept any object as a key.

    :   **Specified by:**: `getCommonPropertyType` in class `ELResolver`
    :   **Parameters:**: `context` - The context of this evaluation.: `base` - The map to analyze. Only bases of type `Map` are handled by this resolver. **Returns:**: `null` if base is not a `Map`; otherwise `Object.class`.


---


|  |  |  |
| --- | --- | --- |
| **Overview** \| **Package** \| **Class** \| **Use** \| **Tree** \| **Deprecated** \| **Index** \| **Help** | |  |
| **PREV CLASS**   **NEXT CLASS** | **FRAMES**    **NO FRAMES**     **All Classes** |  |
| SUMMARY: NESTED \| FIELD \| CONSTR \| METHOD | DETAIL: FIELD \| CONSTR \| METHOD |  |


---
